# Postponement of the Tenth Annual Meeting of the American Society of Dental Surgeons

**Published:** 1849-07

**Authors:** 


### Miscellaneous Notices.

### AUGUST, 1849.

*Postponement of the Tenth Annual Meeting of the American Society of Dental Surgeons.*—Fearing that the meeting, if held at the time and place appointed at the previous convocation of the Society, would, in consequence of the general prevalence of the cholera, be very thinly attended—the President, Dr. Eleazar Parmly, at the solicitation of a number of the members, who would under other circumstances have attended, deemed it advisable to postpone it indefinitely, and accordingly directed the Secretary, Dr. Westcott, to notify the members to that effect. This has been done, and the meeting, therefore, is postponed; but so soon as it shall be thought advisable to hold it, we understand that it is the intention of the President to convene the members, and we doubt not that it will be more numerously attended than it would have been, if it had been held at the time and place appointed for it at the last meeting. The course which the President has seen proper to take in this matter, has, so far as we have been able to ascertain, with a single exception, met with the hearty approval of the members of the Society.—*Balt. Ed.*

